# Early tumour shrinkage as a survival predictor in patients with recurrent glioblastoma treated with bevacizumab in the AVAREG randomized phase II study

**DOI:** 10.18632/oncotarget.15735

**Published:** 2017-02-25

**Authors:** Alba A. Brandes, Gaetano Finocchiaro, Vittorina Zagonel, Michele Reni, Alessandra Fabi, Claudia Caserta, Alicia Tosoni, Marica Eoli, Giuseppe Lombardi, Matteo Clavarezza, Alexandro Paccapelo, Stefania Bartolini, Luigi Cirillo, Raffaele Agati, Enrico Franceschi

**Affiliations:** ^1^ Department of Medical Oncology, Bellaria-Maggiore Hospitals, Azienda USL, IRCCS Institute of Neurological Sciences, Bologna, Italy; ^2^ Molecular Neuro-Oncology Unit, IRCCS Foundation Carlo Besta, Milan, Italy; ^3^ Department of Clinical and Experimental Oncology, Medical Oncology 1, Veneto Institute of Oncology, IRCCS, Padua, Italy; ^4^ Department of Medical Oncology, IRCCS San Raffaele, Milan, Italy; ^5^ Medical Oncology 1, Regina Elena National Cancer Institute, Rome, Italy; ^6^ Oncology Department, Santa Maria Hospital, Terni, Italy; ^7^ Medical Oncology Unit, Ente Ospedaliero Ospedali Galliera, Genova, Italy; ^8^ Department of Neuroradiology, Bellaria Hospital, IRCCS Institute of Neurological Sciences, Bologna, Italy

**Keywords:** glioblastoma, RANO, bevacizumab, fotemustine, ETS

## Abstract

**BACKGROUND:**

Disease assessment for recurrent glioblastoma (GBM) represents a challenge, especially with the use of antiangiogenic agents. Moreover, validated neuroradiological predictors of outcome are lacking. Recently, the concept of early tumor shrinkage (ETS) has been developed to better assess the ability of treatments in determining a rapid and remarkable tumor response.

The aim of the study was to evaluate the role of ETS in predicting survival of GBM patients treated with BEV

**METHODS:**

We examined the radiological data of patients with recurrent GBM treated with bevacizumab (BEV) or fotemustine (FTM) in the randomized phase II AVAREG trial (EudraCT: 2011-001363-46).

Radiologic assessments at first disease assessment (day 46) were used to calculate the relative change in the sum of the products of perpendicular diameters of all measurable lesions determined by either T1 contrast and T2/FLAIR.

**RESULTS:**

In patients treated with BEV, the best ETS cut-off was reduction of 15% with T1 contrast and of 40% with T2/FLAIR. Adopting this cut-off for T1 contrast radiological changes, ETS was a significant predictor of OS for patients treated with BEV (HR = 0.511, 95%CI:0.269-0.971, *p* = 0.040). The cut-off obtained for T2/FLAIR was not significantly correlated with OS (*p* = 0.102), but we found a trend for correlation with survival when considering the variable as continuous (*p* = 0.058).

**CONCLUSIONS:**

ETS evaluating T1 contrast reduction is a helpful predictor of survival in patients with recurrent GBM treated with BEV, and if validated in a larger prospective trial could be a helpful surrogate endpoint.

## INTRODUCTION

The incidence of primary CNS tumors in Europe amounts to about 5/100,000 cases per year with 2% of mortality. Malignant gliomas are about 90% of all the malignant tumors of the CNS, and glioblastoma (GBM) is the most common.

Although important progress has been made in the last few years, the treatment of GBM is still one of the greatest challenges in oncology, and the time to progression after first-line therapy remains short. At recurrence, therapeutic options are few and the outcome in terms of disease control is disappointing.

Moreover, data regarding antiangiogenic agents in GBM are conflicting [1, 2].

In the multicentric noncomparative, randomized phase II AVAREG trial (EudraCT: 2011-001363-46) [3], that evaluated the role of bevacizumab (BEV) or fotemustine (FTM) in recurrent GBM, we obtained similar survival with the two compounds.

To date there are no validated biomarkers to predict the outcome of recurrent glioblastoma treated with standard chemotherapy or with BEV.

MRI interpretation is not easy and it is subjected to inter-individual variability, and surrogacy with survival is weak [4, 5]. Moreover, imaging changes do not invariably lead to an improvement in patients’ clinical status. This leads to limit the use of response rate as a primary endpoint in glioma trials.

In the field of medical oncology, new concepts have been developed in order to assess a rapid and remarkable tumor response with different compounds, and the early tumor shrinkage (ETS) has been one of the most investigated [6–8].

ETS predicts long-term outcome in first-line trials of chemotherapy with anti-VEGF monoclonal antibodies in metastatic colorectal cancer.

The aim of the study was to evaluate the role of ETS in predicting survival of recurrent GBM patients treated with BEV

## PATIENTS AND METHODS

### Eligibility criteria

Patients with measurable disease defined by RANO criteria as bidimensionally contrast enhancing lesions with clearly defined margins by MRI scan, with two perpendicular diameters of at least 10 mm visible on two or more axial slices were eligible for this analysis.

Written informed consent from all patients and approval from the institutional review boards of participating centers was obtained.

### Disease assessment

In the AVAREG trial, the primary endpoint was survival rate at 6 months, to avoid potential imaging biases due to vessel permeability alteration. RANO criteria [9] were adopted for disease evaluation. The assessment was performed by local investigators and by independent central revision.

Although RANO criteria do not establish a cut-off for the detection in T2/FLAIR of progressive disease for nonenhancing lesions, we assumed that a ≥25% increase of nonenhancing lesions in T2/FLAIR can be considered as progressive disease. The first tumor assessment was performed after 46 days (+/- 3 days) from the first administration of the study drug, while the following assessments were performed every 56 days (+/- 3 days) until progression. If there was uncertainty regarding disease progression, the patients continued treatment; a confirmatory MRI assessment had to be performed after a 4 weeks interval. If progression was confirmed, the treatment was stopped and the date of progression was the time at which this issue was first raised.

All patients’ MRI scans used by the investigator for the overall disease assessment were collected and reviewed by a central Independent Review assessment blinded to clinical information.

The current analysis was performed only over MRI images and not considering clinical status and dexametasone doses. MRIs were performed with 1.5 or 3 Tesla. The same type of exploration along the study for the same patient was perform.

### Statistical analysis

ETS was assessed in the central review analysis and was defined as the relative change in the sum of products of perpendicular diameters of all RANO measurable target lesions at the first disease assessment compared to baseline.

T1 contrast enhancing areas (T1 contrast) and T2/FLAIR were both evaluated as potential predictors for survival.

Data are reported as median, range and frequencies. Survival data (median survival times with 95% confidence interval) were computed by the Kaplan-Meier procedure and were analyzed by the Cox proportional hazards model. The hazard ratios (HRs) were computed together with their 95% confidence intervals (95%CIs).

Receiver-operating characteristics (ROC) curves [10] were calculated in order to estimate the accuracy of ETS for T1 with contrast and T2/FLAIR in predicting the overall survival at 6, 9 and 12 months. The area under the ROC curve (AUC) was computed together with the 95%CI. The best cut-off was calculated using the maximization of the Youden's Index [11].

The SPSS (Version 13.0 for Windows; SPSS Inc., Chicago, IL, USA) was used as a statistical package. Two-tailed P values less than 0.05 were considered significant.

## RESULTS

### Patient baseline characteristics

The population assessable for ETS analysis was 75 patients (82.4% of the study population - Tables 1 and 2), 47 patients in the BEV arm and 28 patients in the FTM arm.

**Table 1:** Patients’ characteristics

Characteristic		*N* = 75
Age	Median	59
	Range	28 – 78
Gender	Male	49 (65%)
	Female	26 (35%)
Treatment	BEV	47 (63%)
	FTM	28 (37%)

**Table 2:** Patients excluded from the analysis

	*N*
Treatment interruption for reasons other than PD	5
Death for reason other than GBM	1
No measurable disease at time of randomization	6
No imaging available for central review	4
Total	16

Response rate was 42.6% and 14.3%, in BEV and FTM arms. Median survival was 7.4 (95%CI 6.0-8.9) and 8.6 (95%CI 6.6-10.8) months, respectively, similarly to the entire population of the study [3]. After progression, 59.6% and 67.9% of patients in the BEV and FTM arm, respectively, received a third line treatment. Four patients (14%) in the FTM arm received BEV, and 24 patients (51%) in the BEV arm received FTM or other nitrosoureas.

### Early tumor shrinkage results

All MRIs of the patients have been centrally evaluated by 2 neuroradiologists (RA and LC) and one oncologist (EF) accordingly to RANO criteria.

At first assessment, the median relative change with respect to baseline (median ETS) was a decrease of 33% for T1 contrast enhancement and 20% for T2/FLAIR, in BEV arm.

A median relative change with respect to baseline was an increase of 24% of tumor area for T1 contrast enhancement (median ETS of -24%), and an increase of 2% (median ETS of -2%) for T2/FLAIR, was found in FTM arm.

**Figure 1:**
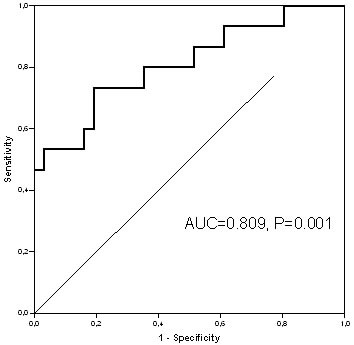
ROC curve for ETS with T1 contrast for the patients treated with BEV

We found that OS6, primary endpoint of the AVAREG study, was the most correlated timepoint for the ROC analysis with ETS (AUC 0.809, *P* = 0.001 for T1 contrast, Figure 1, and AUC = 0.719, *P* = 0.025 for T2/FLAIR in BEV group).

Other evaluated timepoints were OS9 and OS12. OS9 showed ROC AUCs of 0.675 (*P* = 0.049), and 0.610 (*P* = 0.243) for T1 contrast and T2/FLAIR, respectively; OS12 showed ROC AUCs of 0.718 (*P* = 0.026), and 0.606 (*P* = 0.315) for T1 contrast and T2/FLAIR, respectively.

With the aim to assess the best correlation between OS timepoints and ETS, we evaluated the ROC curves and applied the Youden's index.

The best ETS cut-off calculated using the maximization of the Youden's Index was 15% for T1 contrast and 40% for T2/FLAIR.

**Figure 2:**
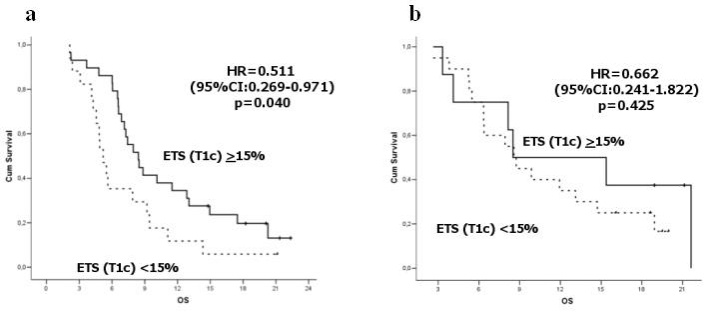
Survival according to ETS (T1 contrast) in BEV arm **a**. and FTM arm **b**.

Adopting the 15% cut-off for T1 contrast radiological changes, ETS was a significant predictor of OS for patients treated with BEV (median OS: 8.4 months *vs* 5.2 months, HR = 0.511, 95%CI:0.269-0.971, *P* = 0.040 - Figure 2).

The cut-off for ETS assessed using T2/FLAIR area variation was not correlated with OS in patients treated with BEV (HR = 0.555, 95%CI:0.274-1.125 *P* = 0.102)

Nonetheless, a trend was found when considering T2/FLAIR area variation as a continuous variable (*P* = 0.058).

We analyzed also the ETS cut-off values in the FTM arm, and we did not found any significant correlation for T1 contrast (*P* = 0.425) and T2/FLAIR (*P* = 0.414).

## DISCUSSION

Traditional imaging presents several limitations to predict the outcome of patients with recurrent GBM.

In particular, the role of response assessment and response rate has been debated since responses are rare, often only short-lived and may be without tangible clinical relevance.

The evaluation and correlation of response with survival end-points has been investigated in a large number of patients with newly diagnosed (*n* = 1359) and recurrent GBM (*n* = 357) treated prospectively on North Central Cancer Treatment Group trials [4]. For recurrent GBM, response was an acceptable surrogate for TTP or PFS, but not for OS. Recently, a landmark analysis of the phase II BRAIN study suggested that response rate should be correlated with OS-12. However, due to the lack of validated response criteria for antiangiogenic treatments in neuro-oncology, the relationship between response and OS has to be better investigated [5, 12]

Many molecular (i.e. proneural pattern [13] or biological features [14]), morphological [15, 16] or novel imaging techniques [17, 18], and mathematical models [19] have been tested to predict the clinical outcome using antiangiogenic agents, but without definitive results.

In fact, the use of these agents alters the blood brain barrier, that raised the issue of pseudoresponses in the field of neuro-oncology [5]. This phenomenon consists of reduction of contrast enhancement due to reduced vascular permeability but does not necessarily reflect a real tumor response. In fact, despite the decrease in contrast enhancement, patients may show a simultaneous increase in the nonenhancing imaging, depicted in T2/FLAIR images.

In order to reduce the impact of pseudoresponses, the recently proposed Response Assessment in Neuro-Oncology (RANO) criteria also consider T2/FLAIR to evaluate the nonenhancing component of the tumor [9].

These criteria have been shown to be strongly concordant with other methods (MacDonald, RECIST, RECIST+FLAIR) in determining response and progression to irinotecan-bevacizumab [20], even if in a retrospective analysis of MRIs in the BRAIN study [21] the consideration of T2/FLAIR hyperintensity led to reduced response rates and to anticipated disease progression identification [22]. At this time RANO criteria are the standard in all new generation of clinical trials.

This step improved the role of imaging-based endpoints such as response rate and PFS and has relevant implications since they can decrease time of performing clinical trials. Moreover, they are not influenced by crossover, and can quantify effects of therapeutic regimens on tumor growth [23].

To confirm this concept, Huang and Colleagues showed that objective response and PFS determined by either RANO or Macdonald criteria correlated with survival [22].

However, further steps are needed and earlier indicators of effectiveness (biochemical, morphological, or functional) could permit a more rapid evaluation of treatment activity, and might help to estimate treatment-efficacy and treatment-impact on prognosis based on the velocity or alteration of tumour regression.

ETS in tumour size at first reassessment, has been recently investigated retrospectively in first-line trials of metastatic colorectal cancer, and appears to be associated with better outcomes [6–8]. Moreover, rapid responses to BEV treatment have been found to be associated with increased PFS in recurrent GBM [24].

We analysed the association between ETS and survival for patients treated in the prospective AVAREG trial, according to RANO criteria. Worth mentioning, these data regarding ETS have been assessed by a central Independent Review Committee blinded to clinical information and only the radiological part of the RANO criteria was evaluated.

ETS was not able to predict survival in patients with FTM. This could be due to the limited number of patients (the AVAREG study was a 2:1 ratio randomized trial), but also to the limited response that is achieved with FTM. In fact, the median variation of the T1 contrast enhancing area at the first assessment was an increase of 24% in tumoral area.

ETS of T1 contrast was significantly associated with increased survival in patients treated with BEV, suggesting that achieving rapid decrease in T1 contrast enhancement may lead to prolonged survival.

We did not find any correlation between ETS measured on T2/FLAIR and survival in patients treated with BEV. Only a trend for correlation (*P* = 0.058) was found when ETS measured on T2/FLAIR was considered as a continuous variable.

Our findings confirm the difficulties in understanding and interpret the changes of T2/FLAIR in patients treated with BEV.

In fact, despite that T2/FLAIR evaluation is required accordingly to RANO criteria to better define response or progression [22] these imaging sequences are influenced by multiple factors such as radiation effects, ischemic injury, infection, seizures, and postoperative gliosis, which can alter T2 relaxation time [9]. Thus, these factors may hamper the T2/FLAIR imaging interpretation since these changes are not relevant to tumor growth. Moreover, T2/FLAIR hyperintensity includes both edema and tumor burden and the different relative decrease of one or both these components could differently influence correlation with survival. Moreover, in a retrospective analysis of radiologic data obtained from the BRAIN trial [21] the correlation between response rate, PFS and OS was not improved if they had been evaluated with RANO criteria that included T2/FLAIR evaluation as compared to Macdonald's criteria [22, 25].

In conclusion, ETS might be a helpful predictor of GBM survival in patients treated with BEV. These findings support that inducing a rapid T1 contrast enhancement shrinkage may translate into a survival advantage, while the role of T2/FLAIR decrease is less clear. These data should be confirmed in a larger study.
